# Occurrence and overlap of natural disasters, complex emergencies and epidemics during the past decade (1995–2004)

**DOI:** 10.1186/1752-1505-1-2

**Published:** 2007-03-01

**Authors:** Paul B Spiegel, Phuoc Le, Mija-Tesse Ververs, Peter Salama

**Affiliations:** 1 UNHCR, Geneva, Switzerland; 2 International Public Health Consultant, Geneva, Switzerland; 3 Chief Immunization Unit, UNICEF, NYC, USA

## Abstract

**Background:**

The fields of expertise of natural disasters and complex emergencies (CEs) are quite distinct, with different tools for mitigation and response as well as different types of competent organizations and qualified professionals who respond. However, natural disasters and CEs can occur concurrently in the same geographic location, and epidemics can occur during or following either event. The occurrence and overlap of these three types of events have not been well studied.

**Methods:**

All natural disasters, CEs and epidemics occurring within the past decade (1995–2004) that met the inclusion criteria were included. The largest 30 events in each category were based on the total number of deaths recorded. The main databases used were the Emergency Events Database for natural disasters, the Uppsala Conflict Database Program for CEs and the World Health Organization outbreaks archive for epidemics.

**Analysis:**

During the past decade, 63% of the largest CEs had ≥1 epidemic compared with 23% of the largest natural disasters. Twenty-seven percent of the largest natural disasters occurred in areas with ≥1 ongoing CE while 87% of the largest CEs had ≥1 natural disaster.

**Conclusion:**

Epidemics commonly occur during CEs. The data presented in this article do not support the often-repeated assertion that epidemics, especially large-scale epidemics, commonly occur following large-scale natural disasters. This observation has important policy and programmatic implications when preparing and responding to epidemics. There is an important and previously unrecognized overlap between natural disasters and CEs. Training and tools are needed to help bridge the gap between the different type of organizations and professionals who respond to natural disasters and CEs to ensure an integrated and coordinated response.

## Introduction

The causes of disasters are not always clear and often overlap. For example, Sen argues that famines are usually caused by a lack of purchasing power or entitlements and not necessarily due to drought and consequent food shortage, which can be exacerbating factors [1]. An epidemic may be controlled easily under certain circumstances and thus not turn into a disaster; however, if the population's ability to respond to the epidemic is reduced due to external factors, such as a natural disaster or complex emergency (CE), then the epidemic may indeed become a disaster (see table 1 for definitions).

There are few articles and data that examine the frequency of occurrence and overlap among natural disasters, complex emergencies and epidemics. These data have important implications for disaster planning and response. Do large-scale epidemics commonly occur following large natural disasters, as was recently loudly claimed by the World Health Organization (WHO) and widely repeated in the media worldwide following the recent Asian tsunami [2,3]? If so, which type of epidemics? If natural disasters frequently occur in areas of a complex emergency, then the skills of the humanitarian workers may need to be broadened to include appropriate planning and response to natural disasters.

The fields of expertise of natural disasters and CEs are quite distinct with different tools for mitigation and response as well as different types of competent organizations and qualified professionals who respond. However, natural disasters and CEs can occur concurrently in the same geographic location and epidemics can occur during or after either event. For example, in the recent Asian tsunami, affected areas in Sri Lanka and Aceh province, Indonesia, have rebel insurgencies, and Somalia has been in civil war for decades [4]. In the Gode district of Ethiopia in 2000, a drought and consequent food crisis, civil strife and a measles epidemic all occurred during the same period and location (see case study) [5].

The objectives of this article are twofold: (1) to identify large-scale natural disasters, CEs and epidemics over the past decade (1995–2004); and (2) to document, for each of the large-scale events in the above three categories, the occurrence in the same location and relevant timeframe of the other two types of events, regardless of their magnitude.

## Methods

The data sources consisted of using the Center for Research on the Epidemiology of Disasters' (CRED) Emergency Events Database (EM-DAT), a database containing essential core data on the occurrence and effects of over 12,800 mass disasters in the world from 1900 to present [6], for natural disasters, the Uppsala Conflict Database, a database that contains information on armed conflicts of the world since 1989 [7], for CEs, and the WHO outbreaks archive, a database that contains information on worldwide outbreaks since 1996 [8], for epidemics. Corroborating data were obtained from PubMed [9], Database on the Human Impact of Complex Emergencies (CE-DAT) [10], LexisNexis news service database [11], Central Intelligence Agency World Factbook [12], and GlobalSecurity.org [13]. The Uppsala conflict database was used instead of CRED's CE-DAT because the primary source of data for natural disasters was from CRED's EM-DAT and the authors wanted to use different primary sources for each major event. However, CE-DAT was used to corroborate the Uppsala data and there were no significant differences. Data were analyzed using EpiInfo 3.2.2 Software [14]. Since the WHO outbreak archive began in 1996, we used the corroborating data sources to document epidemics for 1995.

Only events occurring within the past decade (1995–2004) that met the definitions of a natural disaster, CE, or epidemic for this article (table 1) were included. If there were conflicting data, we prioritized peer-reviewed published literature followed by the main database used for each event. The largest 30 events in each category were based on the total number of deaths recorded; they are referred to in this article as ***large-scale events***. Thirty events were considered to be sufficient to meet the objectives of the article as well as to allow the authors to clarify and resolve conflicts in the data and to match timeframes and geographic location. However, other **concurrent events **that met the inclusion criteria with each major event category were included regardless of the magnitude of mortality. These **other events **were recorded as occurring within each major event if they occurred in a specific timeframe and in the same geographical location but not necessarily among the same populations affected by the events; the data did not allow for such a distinction. The same geographical location refers to the same state or similar type of entity (e.g. province) in a country but not necessarily overlapping among the same population (e.g. occurring in 2 different districts in the state). For example, any epidemics or natural disasters that occurred within the timeframe and location of a large-scale CE, regardless of the number of deaths (e.g. the 2004 Asian tsunami was considered to be linked with a CE because affected areas included CEs in Sri Lanka, Somalia and Indonesia) were included. Natural disasters were linked to a large-scale epidemic if they occurred within six months before the onset of the epidemic and within the same geographic location. Conversely, epidemics were linked to a large-scale natural disaster if they occurred within the following six months after the natural disaster and within the same geographic location. Events that affected many countries, such as the 2004 Asian tsunami, meningococcal epidemics in the African meningitis belt, and the 2003 heat wave in Europe were counted as one event. Terrorism events, such as the 2001 World Trade Center attack, bioterrorism, and human-made disasters, such as transportation and industrial accidents (e.g. Chernobyl, 1986) were not included in the three event categories. Chronic diseases, such as HIV/AIDS and tuberculosis, were excluded from the category of epidemics.

**Table 1:** Definitions

A **disaster **is a serious event that causes an ecological breakdown in the relation between humans and their environment on a scale that requires extraordinary efforts to allow the stricken community to cope, often with outside help or international aid [16, 17]. Disasters are clearly delineated into two major categories – those caused by natural phenomenon and those generated by humans. In natural disasters, a natural hazard impacts a population or area and may result in severe damage, destruction and increased morbidity and mortality that overwhelm local coping capacity [16].

**Natural disasters **can have an acute onset, such as geologic and climatic hazards (e.g. tsunamis, floods, and hurricanes), or slow onset such as drought and desertification. In **complex emergencies **(CEs), also called humanitarian emergencies, are defined as a humanitarian crisis in a country, region or society with total or considerable breakdown of authority resulting from internal or external conflict that requires an international response [31]. In CEs, mortality among the civilian population substantially increases above the population baseline mortality, either as a result of the direct effects of war, or indirectly through the increased prevalence of malnutrition and/or transmission of communicable diseases, especially if the latter result from deliberate political and military policies and strategies [22].

**Epidemics**, defined as an unusual increase in the number of cases of an *acute *infectious disease which already exists in the region or population concerned or the appearance of an infection previously absent from a region [10] can also be a disaster. For the purposes of this article, cases refer to mortality and not morbidity. Epidemics are differentiated from natural disasters, the latter being a physical or geological force of nature rather than biological. They can occur regularly, such as meningococcal meningitis in the meningitis belt of Africa. However, the occurrence of epidemics can increase and/or be exacerbated after natural disasters and CEs.

## Analysis

**Figure 1:**
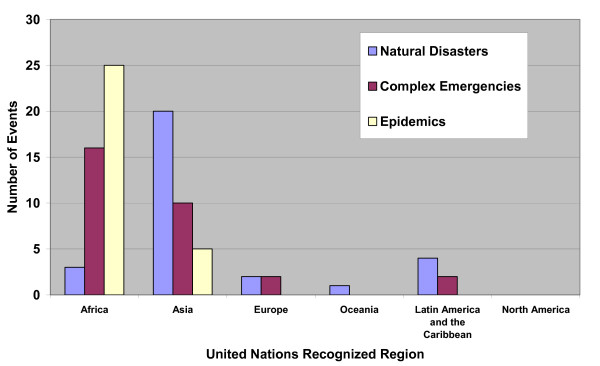
Largest 30 natural disaster, complex emergency and epidemic events based on mortality during the last decade (1995–2004) by region.

**Figure 2:**
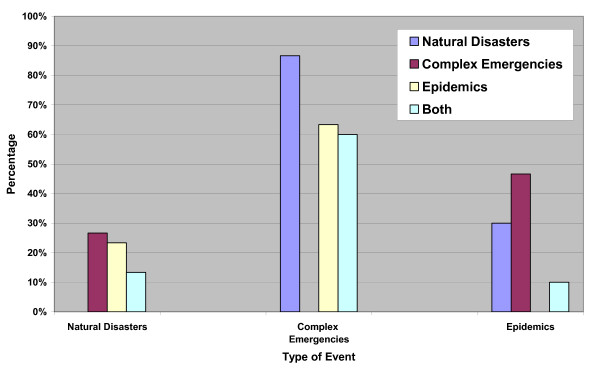
Occurrences of natural disasters, complex emergencies and epidemics during the last decade (1995–2004)*. * For each of the large-scale events in the above three categories, the occurrence in the same location of the other two types of events, regardless of their magnitude or number of events that occurred, was recorded.

Throughout the decade, our research found 3,197 recorded natural disasters, 363 recorded complex emergencies, and 1,374 recorded epidemics. The median duration of the largest 30 natural disasters (table 2) during the past decade was 1 day (0.003 years) with a range of 1 to 2,555 days (0.003 to 7 years). The outlier in these data is North Korea where a famine occurred over a 7 year period. As stated in the analysis, the North Korea disaster could be classified as a CE or a natural disaster; the Emergency Disaster Database classified it as a natural disaster. The overall estimated mortality in the recorded natural disasters ranged from 1,500 deaths to 2.5 million deaths. The majority occurred in Asia (67%) followed by Latin America and the Caribbean (13%; figure 1). The link with any CE was 27%, any epidemic was 23%, and both events^a ^was 13% (figure 2).

**Table 2:** Largest 30 events according to mortality for natural disasters, complex emergencies and epidemics during the last decade (1995–2004) with concurrent events

	**Natural Disasters**	**Complex Emergencies**	**Epidemics**
	*[Date/Country/Type/Mortality]*	*[Date/Country/Mortality]*	*[Date/Country/Type/Mortality]*
	*[Linked CE/Epidemic]*	*[Linked Nat Dis/Epidemic]*	*[Linked Nat Dis/CE]*

**1**	1995–2002; Korea Dem P Rep; Famine; 220,000 to 2,500,000	1965-Present; Colombia; >42,000	1995; Niger; Meningitis; 3,022
		*Nat Dis (Earthquakes, Floods)*	
**2**	1995; Japan; Earthquake; 5,297	1975–2002; Angola; 1,500,000	1996; Nigeria, Niger, Burkina Faso; Meningitis; 8,945
		*Nat Dis (Drought, Floods, Landslides); Epidemic (Meningitis)*	
**3**	1995; Russia; Earthquake; 1,989	1976-Present; Indonesia; >1,600	1996; Nigeria; Cholera; 1,193
		*Nat Dis (Earthquakes, Floods, Tsunami); Epidemic (Arbovirus, Dengue)*	
**4**	1995; India; Flood; 1,479	1980–1999; Peru; >28,000	1996; Zimbabwe; Malaria; 1,311
		*Nat Dis (Floods, Landslides)*	
**5**	1996; China P Rep; Flood; 2,775	1983–2002; Sri Lanka; >64,000	1996; Sudan; Cholera; 700
		*Nat Dis (Floods, Tsunami)*	*CE*
**6**	1997; Iran Islam Rep; Earthquake; 1,728	1989–1998; Iraq; >6,000	1997; Burkina Faso, Ghana, Mali, Niger, Gambia, Senegal, Togo, Benin, Rwanda; Meningitis; 4,498
**7**	1997; Somalia; Flood; 2,311	1989–2003; Liberia; >2,300	1997; Guinea Bissau; Cholera; 781
	*CE; Epidemic (Cholera)*	*Nat Dis (Floods); Epidemic (Cholera, Shigellosis, Yellow Fever)*	*CE*
**8**	1997; Viet Nam; Typhoon; 3,682	1983-Present; Sudan; >3,000,000	1997; Indonesia; Malaria; 550
		*Nat Dis (Floods, Wildfires); Epidemic (Diarrhea, Meningitis)*	*Nat Dis (Drought)*
**9**	1998; Afghanistan; Earthquake; 2,323	1984-Present; Turkey; >30,000	1997; Mozambique; Cholera; 822
	*CE; Epidemic (Arbovirus)*	*Nat Dis (Earthquakes, Floods)*	
**10**	1998; Afghanistan; Earthquake; 4,700	1989-Present; Pakistan; 27,000	1998; Tanzania; Cholera; 2,025
	*CE; Epidemic (Cholera)*	*Nat Dis (Floods)*	*CE*
**11**	1998; India; Heat Wave; 2,541	1989-Present; India; 27,000	1998; Uganda; Cholera; 1,777
		*Nat Dis (Cyclones, Floods)*	*Nat Dis (Floods); CE*
**12**	1998; Papua New Guinea; Tsunami; 2,182	1989-Present; Philippines**; 21,000–25,000	1998; Indonesia; Dengue; 1,449
		*Nat Dis (Floods, Landslides); Epidemic (Cholera)*	*Nat Dis (Drought)*
**13**	1998; India; Cyclone; 2,871	1990–2004; Rwanda; >800,000	1998; Democratic Republic of Congo; Malaria/Cholera; 778
		*Nat Dis (Floods); Epidemic (Cholera, Meningitis)*	*CE*
**14**	1998; China P Rep; Flood; 3,656	1990-Present; Algeria; 100,000–150,000	1998; Tanzania; Malaria; 590
	*Epidemic (Cholera)*	*Nat Dis (Earthquakes, Floods)*	*CE*
**15**	1998; Mali; Famine; 3,615	1991–2002; Sierra Leone; >10,000	1998; India; Cholera; 679
		*Nat Dis (Flood, Windstorm); Epidemic (Arbovirus, Diarrhea, Meningitis)*	*Nat Dis (Cyclone, Heat Wave)*
**16**	1998; India; Flood; 1,811	1991–2003; Burundi; >6,800	1998; Sudan; Diarrheal; 1,373
		*Nat Dis (Floods); Epidemic (Cholera, Malaria)*	*Nat Dis (Flood); CE*
**17**	1998; Honduras, Nicaragua, Guatemala, El Salvador, Costa Rica, Belize; Hurricane; 18,799	1991-Present; Somalia; >60,000	1998; Mozambique; Cholera; 619
	*Epidemic (Cholera, Leptospirosis, Malaria)*	*Nat Dis (Drought, Floods, Tsunami); Epidemic (Cholera, Measles, Meningitis)*	
**18**	1999; Turkey; Earthquake; 17,127	1992–1997; Tajikistan; 21,000	1999; Sudan; Meningitis; 1,600
	*CE*	*Epidemic (Typhoid)*	*Nat Dis (Floods); CE*
**19**	1999; Taiwan (China); Earthquake; 2,264	1993–2003; Afghanistan; >30,000	1999; Kenya; Malaria; 563
		*Nat Dis (Earthquakes, Landslides); Epidemic (Measles, Cholera, Pertussis)*	
**20**	1999; India; Cyclone; 9,843	1994-Present; Democratic Republic of Congo; >3,000,000	2000; Afghanistan; Measles; 1,200
		*Nat Dis (Volcano Eruptions, Floods); Epidemic (Diarrhea, Plague, Measles, Arbovirus, Respiratory illness outbreaks)*	*CE*
**21**	1999; Venezuela; Flood; 30,000	1994-Present; Russia (Chechnya); 20,000–71,000	2000; Chad; Meningitis; 602
		*Nat Dis (Flood)*	*CE*
**22**	2001; India; Earthquake; 20,005	1995, 2002-Present; Cote d'Ivoire; 1,254	2000; Madagascar; Cholera; 1,226
		*Nat Dis (Floods); Epidemic (Cholera, Meningitis, Yellow Fever)*	*Nat Dis (Cyclone)*
**23**	2002; China P Rep; Flood; 1,532	1996-Present; Nepal; 6,400	2001; Burkina Faso, Benin, Central African Republic, Chad, Ethiopia, Niger; Meningitis; 3,338
		*Nat Dis (Floods, Landslides); Epidemic (Encephalitis)*	*CE (Chad)*
**24**	2003; Algeria; Earthquake; 2,266	1995-Present; Uganda; >3,500	2002; Malawi; Cholera; 609
		*Nat Dis (Flood, Drought, Windstorm); Epidemic (Cholera, Ebola)*	*Nat Dis (Drought, Floods)*
**25**	2003; France, Italy, Germany, United Kingdom, Portugal, Netherlands; Heat Wave; 37,451	1997–2002; Chad; >6,000	2002; Burkina Faso, Niger, Nigeria, DRC, Sudan, Guinea, Mali, Senegal, Burundi, Cote d'Ivoire, Benin, Togo, Rwanda; Meningitis; 2,260
		*Nat Dis (Floods); Epidemic (Meningitis)*	*CE (Burundi, Cote d'Ivoire, DRC, Sudan)*
**26**	2003; Iran Islam Rep; Earthquake; 26,796	1998–1999; Guinea-Bissau; 1,700	2002; Madagascar; Influenza; 671
		*Nat Dis (Floods, Drought, Wildfires); Epidemic (Cholera, Meningitis)*	*Nat Dis (Cyclone)*
**27**	2004; Haiti; Flood; 2,665	1998–1999; Yugoslavia (Kosovo); >5,000	2002; Democratic Republic of Congo; Influenza; 2,593
	*CE*		*CE*
**28**	2004; Haiti; Hurricane; 2,754	1998–2000; Ethiopia; 50,000–100,000	2003; Burkina Faso, Niger; Meningitis; 1,253
	*CE*	*Nat Dis (Floods); Epidemic (Meningitis, Yellow Fever)*	
**29**	2004; Philippines; Tropical Storm; 1,619	1998–2000; Eritrea; 50,000–100,000	2004; Burkina Faso, Nigeria; Meningitis; 573
	*CE*		
**30**	2004; Indonesia (Aceh), Sri Lanka, India, Thailand, Maldives, Somalia, Malaysia, Myanmar, Philippines; Tsunami; 280,958	2000–2002; Guinea; >1,000	2004; Indonesia; Dengue; 658
	*CE (Indonesia, Somalia, Sri Lanka); Epidemic (Tetanus)*	*Nat Dis (Flood); Epidemic (Cholera, Yellow Fever)*	*CE*

The median duration of the largest 30 CEs during the past decade (table 2) was 4,563 days (12.5 years) with a range of 365–14,965 days (1 to 41 years). The estimated mortality ranged from 1,000 deaths to 3 million deaths. The majority occurred in Africa (53%) followed by Asia (33%; figure 1). The link with any natural disaster was 87%, with any epidemic was 63%, and with both events was 60% (both events refer to the other two categories of events that occurred during or after the large-scale event but necessarily at the same time or same location).

The median duration of the largest 30 epidemics during the past decade (table 2) was 107 days (0.29 years) with a range of 31 to 397 days (0.08 to 1.09 years). The estimated mortality ranged from 550 deaths to 4,500 deaths. The majority occurred in Africa (83%) followed by Asia (17%; Figure 1). The link with any natural disaster was 30%, with any CE was 47%, and with both events^b ^was 10% (Figure 2).

The need to prepare for the possible occurrence of epidemics following natural disasters [4,15-18] and during complex emergencies [4,17-22] is well documented. However, the data show that epidemics have occurred much more frequently during large-scale CEs than following large-scale natural disasters. During the past decade, 63% of the largest CEs had at least one epidemic compared with 23% of the largest natural disasters. Some possible explanations include the much longer duration of CEs; the preponderance of CEs occurring in Africa, where numerous diseases of epidemic potential exist, poverty is pervasive and poor public services provide favorable environments for epidemics to prosper [23]; increased malnutrition and population movements; and the more effective prevention measures to avert epidemics following natural disasters than CEs possibly due to easier access to affected populations [4]. The data presented in this paper do not support the oft-repeated assertion that epidemics, especially large-scale epidemics, commonly occur following large-scale natural disasters, as was recently loudly claimed by the WHO and widely repeated in the media worldwide following the recent Asian tsunami [2]; historically, this is incorrect.

Although epidemics do not commonly follow large-scale natural disasters, when large-scale epidemics do occur, they often occur during CEs of any magnitude, and to a lesser extent following natural disasters. One-third of the 30 largest epidemics during the last decade occurred on their own; 47% occurred during at least one CE, 30% following at least one natural disaster, and 10% with both events. Thus, governments, United Nations agencies and non-governmental organizations must continue to prepare for the possibility of epidemics following natural disasters and particularly during CEs.

The occurrence of natural disasters and CEs in the same geographic location has not been well studied [4]. Some articles or books have separately examined natural disasters and complex emergencies but have not explored the overlap between the two categories [17,23] Our analysis shows that 27% of the largest natural disasters during the past decade occurred in areas with at least one ongoing CE while 87% of the largest CEs had at least one natural disaster. Thus, there is significant overlap between natural disasters and CEs; this was larger than the authors expected. There is a clear need for training and tools [24] that help to bridge the gap between the different type of organizations and professionals who respond to natural disasters and CEs; these include trainings on the different types of injuries and infectious diseases that occur according to different events and geographical locations which influence preparedness and response strategies as well as initial assessment and monitoring and evaluating tools that take into account both types of events. Similar arguments have been made to bridge the gap between humanitarian response and development programs [25]. This is particularly important in Asia and Africa, where most of the natural disasters and CEs have occurred between 1995–2004.

Early warning systems for natural disasters and epidemics, although technologically challenging and costly, have been shown to be effective [17]. Despite attempts to develop early warning systems for CEs [26], the complexity of the situation and the political will required to act in a timely manner makes their effectiveness unclear. During the 1990s, the International Decade for Natural Disaster Reduction, mitigation emerged as a major strategy for reducing the impact of natural disasters. Such mitigation strategies proved effective but were not implemented uniformly throughout the world and remain under funded, particularly in developing countries [27]. As with early warning systems, mitigation strategies for CEs are more complicated due to the inherent political nature of the situations.

There are a number of limitations in this article. The data show only an ecological association between events and not a cause and effect relationship. The temporal and spatial occurrence of events may not necessarily be related to one another as the data did not allow us to definitively ascertain if they occurred among the same population. If there was a relationship among these events, its effect was not examined. Some events may not have been captured by the databases used. However, whenever possible, we attempted to triangulate the data from different sources. Some disasters were not easily classified into one category and thus misclassification may have occurred; for example, the famine in North Korea could be categorized as a natural disaster or CE; we chose the former. The largest natural disasters, CEs and epidemics during the past decade were arbitrarily limited to the biggest 30 according to mortality; this limited our sample size and does not include other important ways to categorize disasters, such as morbidity and persons affected. Mortality was chosen because it is an essential outcome and the most commonly reported data in the databases used. However, for some types of epidemics and natural disasters, mortality may not be a major outcome and thus morbidity may have been a better outcome to measure the magnitude of these events. Chronic diseases, such as HIV/AIDS and tuberculosis, although causes of major mortality throughout the world, have not been classified as epidemics according to the definition and databases used in this paper.

One strong conclusion of the article is that the longer an event, the higher the risk to have a concomitant event. Since CEs occur over a much longer time period than natural disasters and epidemics, the conclusion that epidemics occur much more commonly during large-scale CEs than following natural disasters is intuitive; however, it is important to have data to support this assertion, which our paper clearly provides. Furthermore, this conclusion has important policy and programmatic implications. Appropriate stockpiling of vaccines, medications and other essential supplies need to be kept up to date and accessible over a long period of time. Since accessibility may be difficult in these emergency situations, proper preparedness planning must occur, including having multiple stockpiles within the same country in order increase the possibility of distributing the supplies. This type of stockpiling must be weighed against the increased cost of having multiple stockpiles in a country. A functioning epidemic alert and response system needs to be established and maintained. Furthermore, the high turnover of staff working in CEs means that continuous training needs to be provided over many years.

### Ethiopia case study

Ethiopia has been subject to recurrent drought and food shortages which have sometimes been exacerbated by civil strife [28,29]. These crises have often resulted in massive excess mortality and population displacement. Beginning in 1999, data from early warning systems in many regions of Ethiopia indicated that the food security and nutrition situation was deteriorating rapidly [30]. The World Food Program estimated that more than 10 million people needed food assistance at the peak of the crisis. The Somali region in Ethiopia was the worst affected; this region is inhabited by predominantly pastoralist and agro-pastoralist communities which are highly vulnerable to changing climactic conditions and are subject to recurrent food security crises. Furthermore seasonal migration is one of the key coping strategies for these communities. The situation in Somali region was exacerbated by insecurity, conflict and poor health infrastructure.

In early 2000, cases of severe malnutrition and measles began to be reported by non-governmental organizations but it was not until April 2000 when media attention began to focus on Gode in Somali region that a large-scale international humanitarian response was triggered. The humanitarian response was not only delayed but was also overly focused on food-based interventions such as the general food ration and therapeutic and supplementary feeding for severely and moderately malnourished individuals-the so called 'food first bias' [5]. While such interventions are critical for preventing and treating malnutrition, by attracting people to population centers in the absence of health-related interventions, these interventions risk contributing to mortality while paradoxically addressing malnutrition. In Gode, the crude mortality rate (CMR) was 3.2/10,000/day or over 6 times the CMR for sub-Saharan Africa. Measles-related mortality was particularly important among remote, rural communities who may not have been exposed to measles wild-virus and have not been reached by immunization services. Such communities do not normally benefit from herd immunity which generally requires a population coverage of more than 90% for measles immunization. The measles epidemic in the conflict-affected and food insecure was severe; measles alone or in combination with acute malnutrition accounted for 22% of deaths among children under 5 years and for 17% of deaths among children 5 to 14 years of age [5].

## Conclusion

Large scale natural disasters and CEs have occurred primarily in Africa and Asia from 1995–2004. Epidemics with mortality have occurred much more frequently during large-scale CEs than following large-scale natural disasters during the past decade. The data presented in this paper do not support the common assertion that epidemics, especially large-scale epidemics, commonly occur following large-scale natural disasters. There is a significant and previously unrecognized overlap between natural disasters and CEs. Training and tools are needed to help bridge the gap between the different type of organizations and professionals who respond to natural disasters and CEs to ensure an integrated and coordinated response. Further study of the relationships among natural disasters, CEs and epidemics is needed to define the extent to which the occurrence of one type of disaster enhances the risk of another.

## Authors' contributions

PBS conceived of the study, designed the research plan, supervised the literature review and data analysis, and wrote the paper. PL participated in the study design, co-wrote the paper, undertook the literature review and data analysis. MTV participated in the study design, co-wrote the paper, undertook the literature review. PS assisted in the critical interpretation of the intellectual content and drafting of the paper.

## Competing interests

The authors and their institutions have no financial or other conflicts of interests. There were no grants or outside funding for this work.
